# Habitat preferences rather than morphological traits affect the recovery process of Collembola (Arthropoda, Hexapoda) on a bare saline–alkaline land

**DOI:** 10.7717/peerj.9519

**Published:** 2020-07-21

**Authors:** Zhen Ni, Xiumin Yan, Liang Chang, Xin Sun, Donghui Wu, Bing Zhang

**Affiliations:** 1 Heilongjiang Bayi Agricultural University, Agronomy College, Daqing, Heilongjiang, China; 2 Heilongjiang Bayi Agricultural University, Research Center of Saline and Alkali Land improvement Engineering Technology in Heilongjiang Province, Daqing, Heilongjiang, China; 3 Guizhou Education University, School of Geography and Resources, Guiyang, Guizhou, China; 4 Institute of Northeast Geography and Agroecology, Chinese Academy of Sciences, Key Laboratory of Wetland Ecology and Environment, Changchun, Jilin, China; 5 University of Göttingen, J.F. Blumenbach Institute of Zoology and Anthropology, Göttingen, Germany; 6 Northeast Normal University, Jilin Provincial Key Laboratory of Animal Resource Conservation and Utilization, Changchun, Jilin, China; 7 Northeast Normal University, Key Laboratory for Vegetation Ecology, Changchun, Jilin, China; 8 Peking University, Key Laboratory for Earth Surface Processes of the Ministry of Education, Institute of Ecology, College of Urban and Environmental Science, Beijing, China

**Keywords:** Springtails, Dispersal, Degraded grassland, Soil transfer, The Songnen Plain

## Abstract

The Songnen Plain of China was once an important grassland used for sheep grazing, but it has largely been degraded to bare saline-alkaline land (BSAL). BSAL consists of plant-free areas characterized by high soil pH values (up to 10) and salt and alkali (e.g., Na^+^ and Ca^2+^) contents, as well as low soil organic matter and water contents; thus, very few soil faunal species can survive on BSAL. The recovery of degraded ecosystems provides a great opportunity to investigate the reconstruction of belowground soil faunal communities. Collembola are a class of widespread and abundant soil fauna that can colonize this harsh environment. Habitat changes on BSAL promote aboveground revegetation, which greatly facilitates the recovery of Collembola. A soil transfer experiment on the BSAL of the Songnen Plain was conducted to study the effects of habitat and Collembola morphological traits on the recovery process of Collembola. Defaunated and with-fauna soil blocks were transferred among three habitats: BSAL, reclaimed arable land, and naturally revegetated grassland. The recovered Collembola in the transferred soil blocks were compared two, seven, and 12 weeks after the start of the experiment. The results showed that (1) the majority of the Collembola, regardless of their morphological traits, recovered in the defaunated soil blocks within 2 weeks; (2) generalists and habitat-preferring species recovered faster than specialists; (3) the average total abundance, species richness, and community composition of Collembola recovered to the natural levels in 2 weeks; and (4) 12 weeks after replacement, the highest average total abundance and species richness of Collembola were found in the arable land. Our results indicate that the majority of Collembola in this study, regardless of their dispersal type, which is related to their morphological traits, are fast dispersers, and their recovery speeds are mainly affected by habitat preferences. We suggest that the reclamation of BSAL to arable land rather than its natural recovery to grassland aids in the recovery of Collembola in degraded grassland systems.

## Introduction

Poor land management practices and environmental change are affecting belowground communities globally, reducing their biodiversity and benefits to human health (Wall, Nielsen & Six, 2015). The Songnen Plain used to be one of the most important grazing and harvesting pastures in China. However, long-term overgrazing and reclamation disturbances have resulted in the formation of bare saline–alkaline land (BSAL) (Wang et al., 2009), causing 778,000 hectares of grassland deterioration (16.59% of the total area) (Zheng & Li, 1993). The alkalization of surface soils caused by the accumulation of Na^+^ and Ca^2+^ (Zhou et al., 2011) has negative effects on plant growth conditions in BSAL (Zheng & Liu, 1990; Gao et al., 1996; Wu & Li, 2003), creating an inhospitable ecological system for aboveground and belowground organisms. Previous studies showed that habitat changes such as revegetation effectively aided in the recovery of soil quality in degraded environments (Zhang et al., 2011; Wang et al., 2012) and that BSAL was partially colonized by plants, forming a mosaic landscape. In contrast, the process of belowground biodiversity recovery, such as that of Collembola, that accompanies the aboveground revegetation process is rarely studied.

Collembola are among the most abundant soil animals, with densities of up to 57,000 ind. m^−2^ in grasslands (Gange & Bower, 1997; Hopkin, 1997). As an integral component of soils, Collembola play a critical role in ecosystem services such as decomposition, nutrient cycling and soil formation (Filser, 2002) as well as affect microbial composition and activity (Rusek, 1998; Behan-Pelletier, 2003). They not only feed on dead organic matter, that is, function as decomposers, but also act as herbivores by consuming plant tissue and fine roots (Hurej, Debek & Pomorski, 1992; Chahartaghi et al., 2005; Endlweber, Ruess & Scheu, 2009). The abundance and species composition of Collembola are significantly impacted by changes in vegetation and soil conditions (Ponge, 1993; Chagnon, Hébert & Paré, 2000; Sabais, Scheu & Eisenhauer, 2011). Thus, the recovery of aboveground plants very likely promotes the recovery of soil-dwelling Collembola.

The recovery of biodiversity in degraded ecosystems depends on the capability of species to reach and occupy different parts of an ecosystem mosaic. Species with a higher dispersal ability and wider habitat preferences are assumed to reach distant patches more quickly and to inhabit a greater number of patches within the landscape. In contrast, species with a lower dispersal ability might not reach certain patches, even those with suitable properties that satisfy the habitat requirements of the species (Dunning, Danielson & Pulliam, 1992; Andrén, Delin & Seiler, 1997). Collembola are small and wingless and are expected to have a limited dispersal ability (Hopkin, 1997). Collembola species with long legs and antennae, a well-developed furcula and a complete visual apparatus, for example, *Entomobrya* and *Tomocerus*, can disperse rapidly, whereas other Collembola species with reduced movement or visual organs, for example, *Mesaphorura*, are considered to have poor dispersal capabilities (Hopkin, 1997; Ponge et al., 2006). Collembola are quick recolonizers after disturbances such as flood, drought, and fire (Lindberg & Bengtsson, 2005; Huebner, Lindo & Lechowicz, 2012; González-Macé & Scheu, 2018). They have been proven to colonize new habitats either passively by wind or actively through locomotion (Dunger, Schulz & Zimdars, 2002) and may disperse at varying rates in different habitats (Auclerc et al., 2009). The aims of the present study were to investigate the effects of morphological traits, such as leg length, antenna and furcula characteristics, and habitat preferences, on the recovery process of Collembola. We conducted experiments by transplanting defaunated soil blocks into three habitats: human-disturbed agricultural arable land, naturally revegetated grassland, and seriously degraded BSAL. We hypothesized that (1) rapid dispersers would recover earlier than slow dispersers in all three habitats; (2) generalists would recover faster than specialists in all three habitats; and (3) the total abundance, species richness, and composition of Collembola would recover earlier in the arable land than in the grassland and BSAL. Identifying the factors that affect the recovery of Collembola may greatly aid in our understanding of the restoration of belowground biodiversity during the recovery of degraded grassland.

## Materials and Methods

### Study site

The experiments were performed from June to September 2012 at the Changling Station of Grassland and Agroecology, Chinese Academy of Sciences (44°33′N, 123°31′E), located in the Songnen Plain grassland of Northeast China, one of three soda saline–alkali patches in the world (Li et al., 2003). The area is characterized by plain topography and a typical mesothermal monsoon climate. The annual precipitation is 477 mm (mainly occurring from July to September), while the annual evaporation capacity is 1,600 mm. Land cover in this area includes grassland interspersed with a mosaic of cropland and woodland; however, certain areas contain bare patches due to severe salt accumulation and alkaline conditions. This grassland is characterized as a semiarid meadow steppe, and the most abundant plant species is *Chloris virgata* (Yang & Zhang, 1992).

Three habitats were chosen to perform the experiments: BSAL (B), grassland (G) and arable land (A). The grassland is damaged and degraded due to long-term overgrazing, and no plants have grown in the BSAL since 1998. Part of the BSAL was restored by fencing, resulting in a grassland dominated by *Chloris virgata* for more than 3 years before the experiment. The arable land was reclaimed from BSAL and used for cropping 5 years prior to the experiment. The soils of the arable land, grassland and BSAL were alkaline, with pH values of 8.8, 9.8 and 10.5, respectively. The BSAL contained less soil organic matter (0.4%) than the arable land (1.2%) and grassland (1.3%) and had a higher soil electrical conductivity (1,715 μS/cm) than the arable land (968 μS/cm) and grassland (92 μS/cm).

### Experimental design, field sampling and laboratory procedures

In June 2012, 60 soil blocks (13 cm diameter × 10 cm depth, collected in litter bags with a 0.83 mm mesh size) were sampled from each habitat in areas without vegetation (180 soil blocks in total). Forty-five soil blocks from each habitat were treated by three freeze-thaw cycles (three cycles of −25 °C for 12 h and 15 °C for 12 h) to eliminate soil fauna (defaunated treatments) and then randomly replaced in these three habitats (15 in each). The remaining 15 blocks were untreated and replaced in their original habitats as controls (with-fauna treatments: WAA, WGG and WBB). Five blocks from each treatment were randomly sampled two, seven and 12 weeks after the replacement. Whole soil blocks were taken to the laboratory to extract soil fauna for 24 h in a Tullgren apparatus with a 40 W bulb. Collembolans were preserved in 95% ethanol and then sorted and identified to the species or morphospecies level according to several keys (Bellinger, Christiansen & Janssens, 1996; Christiansen & Bellinger, 1980; Pomorski, 1998; Yin, 1998).

### Characterization of recovery speed, dispersal type, and habitat preference

The recovery speed of each Collembola species was classified into one of four levels (1–4) in each habitat, according to the time when the species reappeared in the defaunated blocks placed back in their original habitats (OAA, OGG and OBB treatments): within 2 weeks (A1, G1, or B1), after 2 and before 7 weeks (A2, G2, or B2), after seven and before 12 weeks (A3, G3, or B3), and did not reappear (A4, G4, or B4). The dispersal types of Collembola were distinguished according to Ponge et al. (2006): species with long legs and antennae, a well-developed furcula and a complete or a small reduction of visual apparatus were considered rapid dispersers, whereas other species with a reduction in mobility or visual organs were considered slow dispersers.

The habitat preference of each species of Collembola was characterized using the IndVal index (Dufrêne & Legendre, 1997), which combines the specificity of a species for a habitat type (maximized when the species is present in only a given habitat type) and its fidelity to this habitat type (maximized when the species is present in all samples of the habitat type). The index is calculated as Ind_*ij*_ = *A*_*ij*_ × *B*_*ij*_ × 100, where *A*_*ij*_ is the average abundance of species *i* in samples of habitat *j* divided by the sum of the species abundances of species *i* in all habitats and *B*_*ij*_ is the number of samples of habitat *j* where the species *i* is present divided by the total number of samples of habitat *j*. Ind_*ij*_ ranges from 0, when species *i* is absent from habitat *j*, to 100, when species *i* is present in all samples of habitat *j* and absent in all other habitat samples. Due to the low abundance in BSAL samples, we obtained only two IndVal values for each species of Collembola, that is, IndA and IndG, indicating a habitat preference for the arable land and grassland, respectively. Classes of habitat preference were then determined using the IndVal values IndG and IndA for each species. Species present in both habitat types and having an IndG/IndA ratio (or the inverse) greater than or equal to 0.25 were classified as “generalists”. Species with an IndA/IndG ratio less than 0.25 were classified as “grassland-preferring species”, and species with an IndA/IndG ratio of 0 were classified as “grassland specialists”. Species with an IndG/IndA ratio less than 0.25 were classified as “arable land-preferring species”, and species with an IndG/IndA ratio of 0 were classified as “arable land specialists” (sensu Auclerc et al., 2009 and Heiniger et al., 2015).

### Statistical analysis

All statistical analyses were performed using the *car* and *vegan* packages in R software (R Development Core Team, 2019). The abundances of Collembola were log (*n* + 1) transformed to meet the assumptions of normality. We used analysis of variance (ANOVA) to test for effects of defaunated soil type compared with the CK on the species richness and abundances of Collembola in the arable land, grassland and BSAL at the three sampling times. When the effect was significant (α = 0.05), we used Tukey’s honestly significant difference (HSD) post hoc test to examine the differences between the defaunated soil types and the CK. We used permutational multivariate analysis of variance (PERMANOVA) to compare the community compositions of Collembola in with-fauna (CK treatments) and defaunated soil blocks in order to reveal the recovery process of Collembola. Principal coordinate analysis (PCoA) based on the Bray-curtis distances was performed using the cmdscale function from the *vegan* package to determine differences among samples of different treatments. The raw data and R script for ANOVA and PERMANOVA are provided in Table S1 and Text S2.

## Results

### Effects of morphological traits and habitat preferences on the recovery speed of Collembola

A total of 13,519 individuals of Collembola from 180 soil blocks were identified as belonging to seven genera and 14 species/morphospecies (Table 1; Table S1). Eleven species were classified as rapid dispersers, and three as slow dispersers. In total, eleven species colonized defaunated soil blocks 2 weeks after replacement, and ten, eight, and five species were classified as A1, G1, and B1, respectively. No clear relationship was found between dispersal type and recovery speed. The three rapid dispersers *Heterosminthurus* sp. 1, *Ptenothrix* sp. 1, and *Entomobrya* sp. 2 were classified as A2-4/G2-4, while three slow dispersers were classified as A1/G1 (except *Isotomiella* sp. 1, which was classified as G2, and *Friesea* sp. 1, which was classified as B2; Table 1).

**Table 1:** The dispersal speeds, dispersal types and habitat preferences of 14 Collembola species.

Species name	Dispersal speed in the			Dispersal type	Habitat preference
	Arable land	Grassland	BASL		
*Arrhopalitidae*					
*Arrhopalites* sp. 1	A1	G3	B2	Slow	Gsp
*Arrhopalites* sp. 2	A1	G2	B4	Slow	Gen
*Bourletiellidae*					
*Heterosminthurus* sp.1	A2	G4	B4	Rapid	–
*Bourletiella* sp.1	A1	G1	B4	Rapid	Gen
*Dicyrtomidae*					
*Ptenothrix* sp. 1	A3	G4	B4	Rapid	Asp
*Ptenothrix* sp. 2	A4	G1	B4	Rapid	–
*Entomobryidae*					
*Entomobrya* sp. 1	A1	G1	B1	Rapid	Gen
*Entomobrya* sp. 2	A3	G2	B3	Rapid	Asp
*Lepidocyrtus* sp. 1	A1	G1	B4	Rapid	Gen
*Orchesellides* sp. 1	A1	G1	B1	Rapid	Gen
*Isotomidae*					
*Proisotoma* sp. 1	A1	G1	B1	Rapid	Apr
*Isotomiella* sp. 1	A1	G2	B1	Slow	Apr
*Neanuridae*					
*Friesea* sp.1	A1	G1	B2	Slow	Apr
*Tullbergiidae*					
*Mesaphorura* sp. 1	A1	G1	B1	Slow	Gen

**Note:**

A1/G1/B1, species that colonized the arable land/grassland/BSAL defaunated blocks within 2 weeks; A2/G2/B2 species that colonized the arable land/grassland/BSAL defaunated blocks after 2 weeks and within 7 weeks; A3/G3/B3, species that colonized the arable land/grassland/BSAL defaunated blocks after 7 weeks and within 12 weeks; A4/G4/B4, species that did not colonize the arable land/grassland/BSAL defaunated blocks over 12 weeks. Dispersal type: rapid = species with long legs and antenna, well-developed furca and complete visual apparatus were considered as having good dispersal capabilities; slow, the remaining species with a reduction in motion or vision organs were considered to have poor dispersal capabilities. Habitat preference: Gsp, grassland specialist; Gen, Generalist; Asp, arable land specialist; Apr, arable land preferring species; “–”, not enough data.

We classified these 12 species as six generalists, three arable land-preferring species, two arable land specialists and one grassland specialist; *Heterosminthurus* sp. 1 and *Ptenothrix* sp. 2 were not analyzed due to low abundances (Table 1). Overall, generalists and habitat-preferring species recovered earlier than habitat specialists: all six generalists and three arable land-preferring species were classified as A1, while two arable land specialists were classified as A3, five generalists were classified as G1, and the grassland specialist *Arrhopalites* sp. 1 was classified as G3 (Table 1).

### Effects of defaunated soil type on the recovery of Collembola

**Figure 1:**
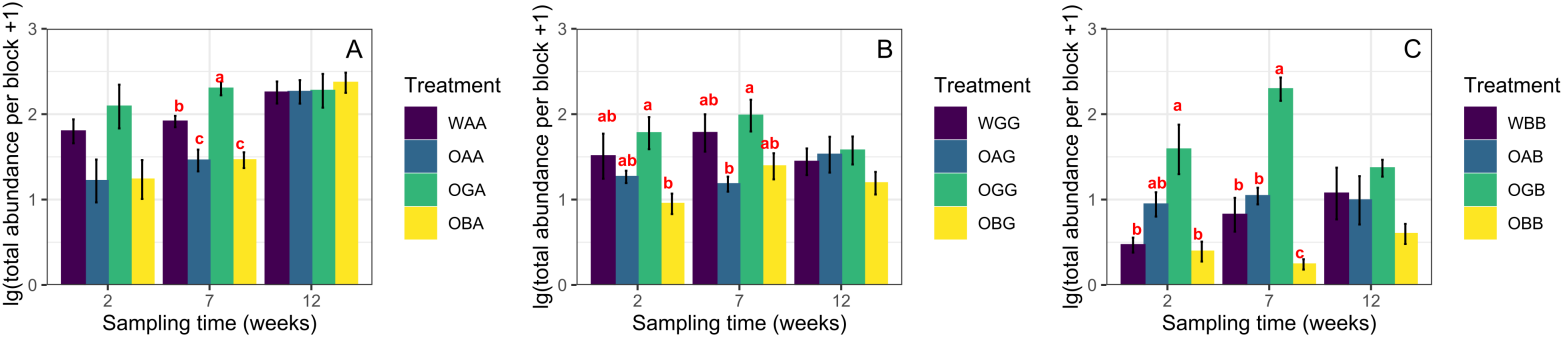
Log transformations of the total abundance (mean ± standard error) of Collembola in control treatments (WAA, WGG and WBB) and recovered to defaunated arable, grassland and BSAL soil blocks replaced to the arable land (A); grassland (B); and BSAL (C). Different lowercase letters above the bars indicate a significant effect among the treatments (HSD post hoc test, *p* < 0.05).

**Figure 2:**
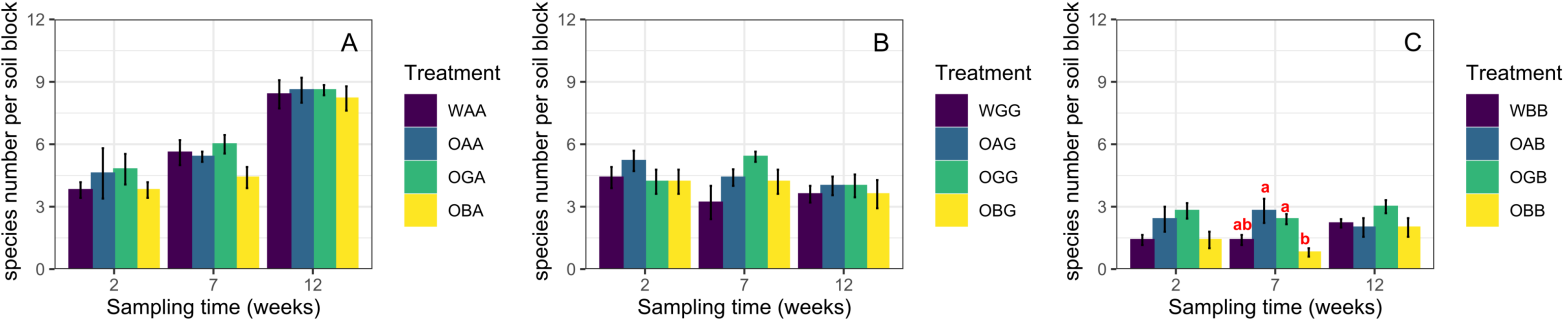
The species richness (mean ± standard error) of Collembola in control treatments (WAA, WGG and WBB) and recovered to defaunated arable, grassland and BSAL soil blocks replaced to arable land (A); grassland (B); and BSAL (C). Different lowercase letters above the bars indicate a significant effect among the treatments (HSD post hoc test, *p* < 0.05).

Two weeks after replacement, the total abundance and species richness of Collembola in defaunated soil were not significantly different from those in the with-fauna treatments (CK) in any of the three habitats (One-way ANOVA, *p* > 0.05, Fig. 1, except significant difference in total abundance between OBB and WBB). Seven weeks after replacement, the average total abundances in OGA/OGB were significantly higher than those in OAA/OAB and OBA/OBB when placed in the arable land and BSAL, respectively (Figs. 1A and 1C); in contrast, no significant difference in species richness was found between the three defaunated soil blocks (except for significantly higher species richness in OAB and OGB than in OBB when placed in the BSAL, Fig. 2C). Twelve weeks after replacement, no significant difference in total abundance or species richness was found between the three defaunated soil types (One-way ANOVA, *p* > 0.05, Figs. 1 and 2).

**Figure 3:**
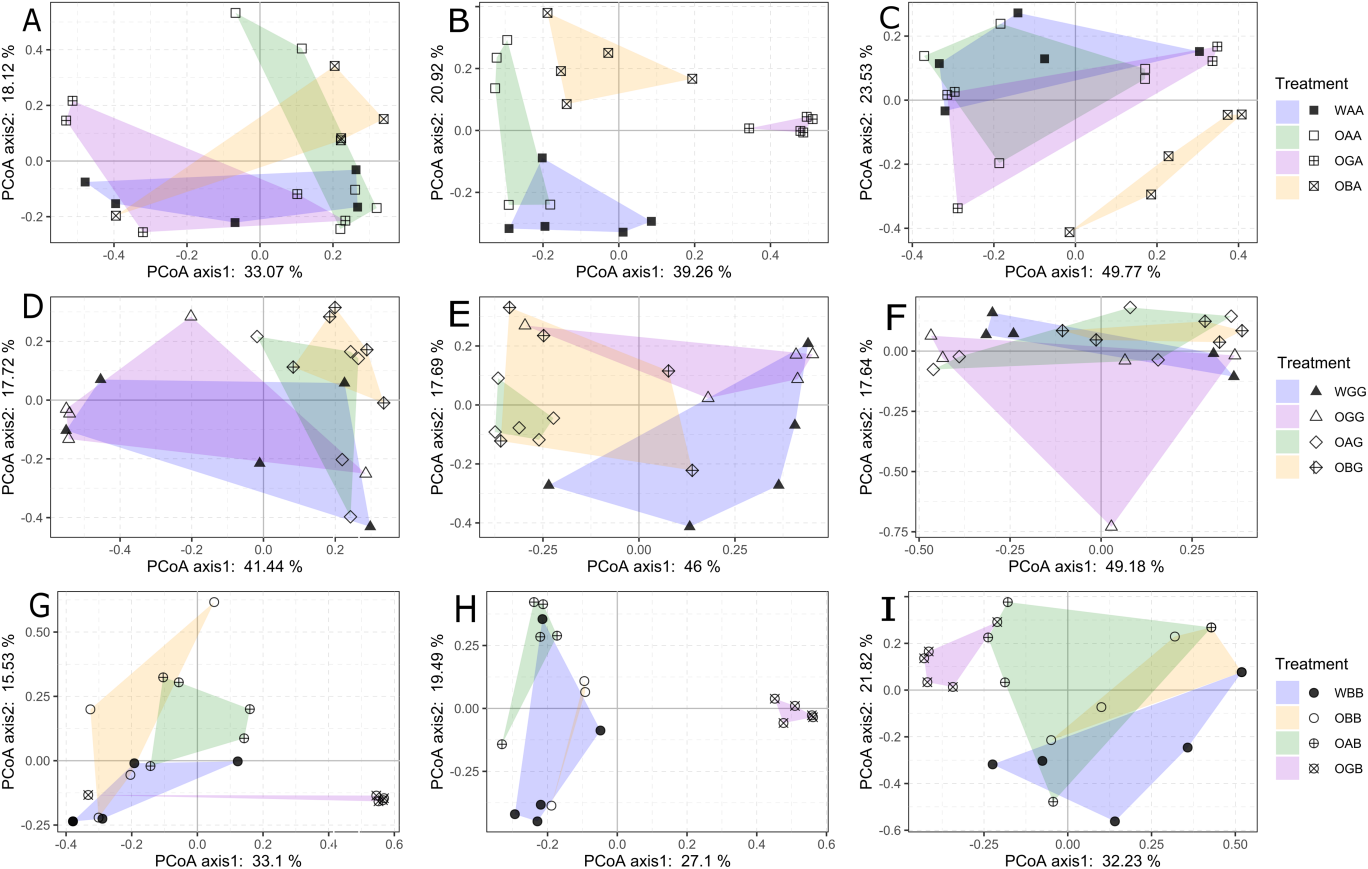
Principal coordinates analysis of Collembola communities between with-fauna treatments (WAA, WGG and WBB, solid points in blue filling polygon) and defaunated soil blocks (OA, OG, and OB) that replaced to the arable land (A–C); grassland (D–F); and BSAL. The analysis was performed separately at each of the three sampling times: 2 (A, D and G), 7 (B, E and H), and 12 weeks (C, F and I) after the replacement. The variance explained by two axes were given for each analysis. Results of permutational multivariate analysis of variance (PERMANOVA) among these treatments were given in Table S3.

In the arable land, the community compositions of Collembola in the three types of defaunated soil (treatment OAA, OGA, and OBA) were not significantly different from that in WAA (the CK treatment) 2 weeks after replacement (PERMANOVA, *p* > 0.05, Fig. 3A; Table S3); but WAA was significantly different from other treatments 7 weeks after the replacement (Fig. 3B; Table S3). In the grassland, the community composition of Collembola in WGG (the CK treatment) was significantly different from that in OBG (*p* = 0.038) but was not significantly different from that in OAG and OGG 2 weeks after replacement (PERMANOVA, *p* > 0.05, Fig. 3D; Table S3); WGG was significantly different from OAG and OBG but not OGG 7 weeks after replacement (PERMANOVA, *p* = 0.01, 0.044, and 0.173, respectively); WGG was not significantly different from other treatments 12 weeks after replacement (PERMANOVA, *p* > 0.05, Figs. 3E and 3F; Table S3). In the BSAL, the community composition of Collembola in WBB (the CK treatment) was significantly different from OAB and OGB (PERMANOVA, *p* = 0.017 and *p* = 0.027, respectively) but was not significantly different from OBB (PERMANOVA, *p* = 0.168, Fig. 3G; Table S3) 2 weeks after the replacement; WBB was significantly different from OGB (*p* = 0.005) but not significantly different from OAB and OBB 7 weeks after replacement (PERMANOVA, *p* > 0.05, Fig. 3H; Table S3).

## Discussion

### Habitat preference rather than morphological traits affects the recovery speed of Collembola

In this study, the recovery speed of each Collembola species was related to its habitat preference rather than to its dispersal type; thus, the first hypothesis was rejected, while the second hypothesis was supported. The majority of Collembola species were classified as A1/B1, indicating that dispersal type based on morphological traits does not allow prediction of the recovery speed of Collembola. Species that are thought to disperse slowly (Ponge et al., 2006), for example, species of *Neanuridae*, and *Tullbergiidae*, even recovered earlier than those that are assumed to be rapidly dispersing species, such as species of *Dicyrtomidae*. It has been shown that slowly dispersing species can also colonize defaunated soil blocks in 1 week (Auclerc et al., 2009). Other studies also showed that both presumed slow and fast dispersers, that is, *Tullbergiinae* (short legs and antennae, no furcula, and no eyes) and *Sminthuridae* (opposite features) of Collembola, were fast dispersers (Ojala & Huhta, 2001). We assume that the dispersion in Collembola or soil fauna in general is driven by the expansion of the population in its distribution area instead of the individual movement. The synchronous abundances of each Collembola species between the with-fauna and defaunated treatments at each time point (Table S4) indicate that the population size of each Collembola species in surrounded habits played important roles in their recovery to defaunated soil blocks. Passive dispersion of Collembola by wind via the soil surface is also possible (Dunger, Schulz & Zimdars, 2002). However, Collembola classified as slow disperser in this study are usually found at the deeper layers of the soil, therefore less susceptible to wind dispersal. Our study also showed that Collembola varied in their recovery speeds according to their habitat preferences and the habitats they inhabited. In the arable land and grassland, generalists and habitat-preferring species usually colonized defaunated soil blocks faster than habitat specialists. This agrees with the finding of a former study that generalists of Collembola are all fast dispersers (Auclerc et al., 2009). An explanation for this result is that generalists are widely distributed in and adapted to both arable land and grassland, therefore, the expansion of the distribution area of their population is more likely to happen than specialists with small populations and restricted distributions.

### The effects of habitat on the recovery of Collembola

The total abundance, species richness, and community composition of Collembola were not significantly different between the defaunated and with-fauna treatments 2 weeks after replacement in any of the three habitats (except for a significantly higher abundance in OGB than in WBB, which likely due to the bait effect of defaunated grassland soil in the BSAL). This indicates that Collembola can recover to the natural level (represented by the with-fauna treatments) in 2 weeks. This speed was independent from the habitats they inhabited, which rejected our third hypothesis. Grassland soil blocks provided the best environment for the recovery of Collembola, in which they showed the highest average total abundance in all three habitats 2 and 7 weeks after replacement. This may be explained by differences in the amount of microbial biomass, the food resource of Collembola, among the three types of defaunated soil blocks, which was disrupted by freeze-thaw cycles (Yanai, Toyota & Okazaki, 2004; Bölter et al., 2005). Defaunated grassland soil blocks replaced in the BSAL and arable land were acting as baits, attracting and gathering most of the specimens which otherwise would be dispersed in the habitats. The sharp decrease in total abundance from seven to 12 weeks in the OGB treatment may have been due to the exhaustion of soil organic matter. Defaunated soil block type did not have a significant effect on the recovery of average species richness in the arable land and grassland, while in the BSAL, more species had colonized the defaunated arable and grassland soil blocks than had colonized the BSAL soil blocks 2 and 7 weeks after replacement, indicating that the recovery process was also strongly limited by the surrounding environment, that is, habitat type.

Arable land provided the best habitat for the recovery of Collembola, in which they showed the highest average total abundance and species richness 12 weeks after replacement. Agricultural practices can increase microbial growth (Petersen et al., 2003), thus providing more food resources than in the grassland and BSAL. In addition, dominant species (>10% of total abundance) in the control treatments played critical roles in the recovery process, especially 2 weeks after replacement. Most dominant species found in the control treatments in the arable land dispersed to defaunated soil blocks within 2 weeks and at high abundances, and the strong variation in total abundance was caused by only a few species, for example, decreases in the abundance of *Orchesellides* sp. 1, *Lepidocyrtus* sp. 1, and *Mesaphorura* sp. 1 in OGG and *Proisotoma* sp. 1 in OGB from seven to 12 weeks after replacement (Table S4).

### Recovery of Collembola in the BSAL

Our study showed that Collembola can recover to natural levels within 2 weeks, reinforcing the assumptions that microarthropods respond rapidly to (Chauvat, Zaitsev & Wolters, 2003) and recover quickly from environmental changes (Lindberg & Bengtsson, 2006; González-Macé & Scheu, 2018). However, in contrast to a previous study showing that surface-living (rapidly dispersing) Collembola recovered more often than species that lived at greater depths (Malmström, 2012), we found that the recovery ability of Collembola was related to their habitat preferences and environment rather than their dispersal type. In the 12-week experiment, the total abundance and species richness of Collembola in the control treatments in the BSAL were significantly lower (2–30 individuals/soil block and 1–2 species) than those in the arable land and grassland, clearly indicating that the BSAL was not able to maintain high belowground biodiversity (Li, Yin & Sun, 2014). However, transplanting defaunated BSAL soil blocks in arable land significantly increased total abundance and species richness (up to 270 individuals/soil block and eight species). Planting vegetation in arable land may promote re-establishment of the associated native arthropod community (Palmer, Ambrose & Poff, 1997), specifically in the BSAL of the Songnen Plain (Wu, Yin & Yin, 2008; Wu, Zhang & Chen, 2005). Collembola herbivores may benefit directly from revegetation (Endlweber, Ruess & Scheu, 2009). The revegetation of the original dominant species (*Leymus chinensis* Tzvel) on the BSAL is very slow under natural conditions (Li & Zheng, 1997). The reclamation of BSAL to arable land, however, can accelerate revegetation processes and change soil conditions, thus providing a more suitable environment for maintaining the diversity and total abundance of Collembola, especially for the three arable land preferring species and a few generalists such as *Bourletiella* sp. 1, *Mesaphorura* sp. 1, and *Orchesellides* sp. 1 (Table S4). Further, the highest species richness and total abundance of Collembola occurring in the arable land suggests that reclamation to arable land rather than natural restoration to grassland may better promote the recovery of Collembola in this degraded grassland system.

## Conclusions

In the field experiment presented herein, we clearly proved that the majority of Collembola species can colonize defaunated soil blocks within 2 weeks, regardless of their dispersal type, which is related to their morphological traits. Generalists and habitat-preferring species recovered faster than habitat specialists. The fast recovery ability of Collembola significantly contributed to the recovery of their total abundance, species richness, and community composition. In the longer term, arable land significantly facilitated the recovery of both the average total abundance and species richness of Collembola, especially for arable land preferring species as well as a few generalists. Thus, in general, the reclamation of BSAL to arable land rather than its natural recovery to grassland may better promote the recovery of Collembola in degraded grassland systems.

## Supplemental Information

10.7717/peerj.9519/supp-1 Supplemental Information 1 The abundance of each Collembola species and species richness in each treatment. Treatments comprise defaunated soil blocks and with-fauna (CK treatments, WAA, WGG, and WBB) that replaced in three habitats. Three letters were used to label the treatments with fauna (W) or defaunated (O) soil blocks, and the second and third letter indicate the source and destination habitats, respectively (A = arable land, B = BSAL, and G = grassland). Destination A, G, and B indicate arable land, grassland, and BSAL. Arr, Arrhopalites; Bou, Bourletiella; Ent, Entomobrya; Fri, Friesea; Het, Heterosminthurus; Iso, Isotomiella; Lep, Lepidocyrtus; Mes, Mesaphorura; Orc, Orchesellides; Pro, Proisotoma; Pte, Ptenothrix. Raw data (named after "Coll_recov_all180_nz.csv") as the input file for R analysis.

10.7717/peerj.9519/supp-2 Supplemental Information 2 R scripts for all analyses.

10.7717/peerj.9519/supp-3 Supplemental Information 3 Results of PERMANOVA between defaunated soil blocks and with-fauna (CK treatments, WAA, WGG, and WBB) that replaced in three habitats. Three letters were used to label the treatments with fauna (W) or defaunated (O) soil blocks, and the second and third letter indicate the source and destination habitats, respectively (B = bare saline-alkaline land, G = grassland, and A = arable land). Significant levels are marked as *, p<0.05; **, 0.01

10.7717/peerj.9519/supp-4 Supplemental Information 4 Total species and abundance of Collembola recovered in five soil blocks.

## Additional Information and Declarations

### Data Availability

The following information was supplied regarding data availability: The raw measurements are available in Table S1.
